# The spectrum and some subdivisions of the spectrum of discrete generalized Cesàro operators on $\ell_{p}$ ($1< p<\infty$)

**DOI:** 10.1186/s13660-017-1464-2

**Published:** 2017-08-22

**Authors:** Mustafa Yıldırım, Nuh Durna

**Affiliations:** Department of Mathematics, Faculty of Science, Cumhuriyet University, Sivas, 58 410 Turkey

**Keywords:** 40H05, 40C99, 46A35, 47A10, spectrum, fine spectrum, Cesàro operator, discrete generalized Cesàro operators

## Abstract

The discrete generalized Cesàro matrix $A_{t}= ( a_{nk} ) $ is the triangular matrix with nonzero entries $a_{nk}=t^{n-k}/ ( n+1 )$, where $t\in [ 0,1 ] $. In this paper, boundedness, compactness, spectra, the fine spectra and subdivisions of the spectra of discrete generalized Cesàro operator on $\ell_{p}$ ($1< p<\infty$) have been determined.

## Introduction

The lower triangular matrix $A_{t}= ( c_{nk} ) $ defined by $c_{nk}=t^{n-k}/(n+1)$, $0< t\leq1$ is called a discrete generalized Cesàro operator. The matrix reduces to the Cesàro matrix by setting $t=1$. In 1982, Rhaly [1] showed that the discrete generalized Cesàro operator $A_{t}$ on the $\ell_{2}$ Hilbert space was a bounded compact linear operator and computed its spectrum. Also in [2], lower bounds for these classes were obtained under certain restrictions on $\ell _{p}$ ($1< p<\infty $) by Rhoades. In this article, we show that this operator is a compact linear operator, calculate its spectrum and get two subdivisions of this spectrum on the $\ell_{p}$ ($1< p<\infty $) sequence space.

## Boundedness of discrete generalized Cesàro operator

In 1982, Rhaly [1] showed that the discrete generalized Cesàro operator $A_{t}$ on the Hilbert space $\ell_{2}$ is a bounded linear operator. We will show that $A_{t}$ is a bounded linear operator on $\ell_{p}$ ($1< p<\infty $).

### Theorem 1

[3] (Hardy inequalities)

*If* $p>1$, $a_{n}\geq0$, *and* $A_{n}=a_{1}+a_{2}+\cdots+a_{n}$, *then unless all* $a_{n}$ *’s are* 0,

2.1 $$ \sum \biggl( \frac{A_{n}}{n} \biggr) ^{p}< \biggl( \frac{p}{p-1} \biggr) ^{p}\sum a_{n}^{p} $$

*inequality is provided*. *This constant is the best possible*.

### Theorem 2

$A_{t}\in B ( \ell_{p} ) $ *and* $\Vert A_{t} \Vert _{B ( \ell_{p} ) }\leq\frac {p}{p-1}$ *for* $0< t<1$, *where* $1< p<\infty$.

### Proof

Using Theorem 1, since $0< t<1$, we have

$$ \begin{aligned} \Vert A_{t}x \Vert _{p}^{p} & =\sum_{n=0}^{\infty } \vert y_{n} \vert ^{p}=\sum_{n=0}^{\infty} \Biggl\vert \frac{1}{n+1}\sum_{k=0}^{n}t^{n-k}x_{k} \Biggr\vert ^{p} \\ & \leq\sum_{n=0}^{\infty} \Biggl( \frac{1}{n+1}\sum_{k=0}^{n} \vert t \vert ^{n-k} \vert x_{k} \vert \Biggr) ^{p} \\ & \leq\sum_{n=0}^{\infty} \Biggl( \frac{1}{n+1}\sum_{k=0}^{n} \vert x_{k} \vert \Biggr) ^{p} \\ & \leq \biggl( \frac{p}{p-1} \biggr) ^{p}\sum _{n=0}^{\infty } \vert x_{n} \vert ^{p}= \biggl( \frac{p}{p-1} \biggr) ^{p} \Vert x \Vert _{p}^{p}.\end{aligned} $$

Hence we get

$$ A_{t}\in B ( \ell_{p} ) \quad\text{and}\quad \Vert A_{t} \Vert \leq\frac{p}{p-1}. $$

 □

## Compactness of discrete generalized Cesàro operator

Compact linear operators have a great deal of application in practice. For instance, they play a central role in the theory of integral equations and in various problems of mathematical physics.

Disentangling the historical development of the spectral theory of compact linear operators is particularly hard because many of the results were originally proved early in the twentieth century for integral equations acting on particular Banach spaces of functions. These operators behave very much like familiar finite dimensional matrices without necessarily having finite rank. For a compact linear operator, spectral theory can be treated fairly completely in the sense that Fredholm’s famous theory of linear integral equations may be extended to linear functional equations $Tx-\lambda x=y$ with a complex parameter *λ*. This generalized theory is called the *Riesz-Schauder theory*.

### Definition 1

[4]

Let *X* and *Y* be normed spaces. An operator $T:X\rightarrow Y$ is called a compact linear operator (or completely continuous linear operator) if *T* is linear and if, for every bounded subset *M* of *X*, the image $T ( M ) $ is relatively compact, that is, the closure $\overline{T ( M ) }$ is compact.

From the definition of compactness of a set, we readily obtain a useful criterion for the operator.

### Theorem 3

[4]

*Let* *X* *and* *Y* *be normed spaces and* $T:X\rightarrow Y$ *be a linear operator*. *Then* *T* *is compact if and only if it maps every bounded sequence* $( x_{n} ) $ *in* *X* *onto a sequence* $( Tx_{n} ) $ *in* *Y* *which has a convergent subsequence*.

The following theorem makes it easy to show the compactness of a linear operator over a normed space.

### Theorem 4

[4]

*Let* *X* *and* *Y* *be normed spaces and* $T:X\rightarrow Y$ *be a linear operator*. *Then*:

- *If* *T* *is bounded and* $\dim T ( X ) <\infty$, *the operator* *T* *is compact*.
- *If* $\dim X<\infty$, *the operator* *T* *is compact*.

The following is important as a tool for proving compactness of a given operator as the uniform operator limit of a sequence of compact linear operators.

### Theorem 5

[4]

*Let* $( T_{n} ) $ *be a sequence of compact linear operators from a normed space* *X* *into a Banach space* *Y*. *If* $( T_{n} ) $ *is uniformly operator convergent*, *say*, *if* $\Vert T_{n}-T \Vert \rightarrow0$, *then the limit operator* *T* *is compact*.

In 1982, Rhaly [1] showed that the discrete generalized Cesàro operator $A_{t}$ on the Hilbert space $\ell_{2}$ is a compact linear operator. We show that $A_{t}$ is a compact linear operator on $\ell _{p}$ ($1< p<\infty $).

### Theorem 6

$A_{t}$ *is a compact linear operator over* $\ell_{p}$ ($1< p<\infty $) *for* $0< t<1$.

### Proof

Let

$$ A_{t}^{r} ( x ) = \Biggl( x_{0}, \frac{1}{2} ( tx_{0}+x_{1} ) ,\frac{1}{3} \bigl( t^{2}x_{0}+tx_{1}+x_{2} \bigr) , \ldots,\frac{1}{r+1} \sum_{k=0}^{r}t^{r-k}x_{k},0,0, \ldots \Biggr) . $$

For $\forall r\in \mathbb{N} $, we obtain that $\dim ( A^{r} ) =r+1<\infty$. Hence, from Theorem 4, for all $r\in \mathbb{N} $, the operator $A^{r}$ is compact on $\ell_{p}$. With triangular inequality and Hölder’s inequality, for all $x\in\ell_{p}$, we have

3.1 $$ \begin{aligned}[b] \bigl\Vert \bigl( A_{t}^{r}-A_{t} \bigr) ( x ) \bigr\Vert _{p}^{p} & =\sum _{n=r+1}^{\infty} \Biggl\vert \frac{1}{n+1} \sum _{k=0}^{n}t^{n-k}x_{k} \Biggr\vert ^{p} \leq\sum_{n=r+1}^{\infty} \Biggl\{ \frac{1}{n+1}\sum_{k=0}^{n}t^{n-k} \vert x_{k} \vert \Biggr\} ^{p} \\ & \leq\sum_{n=r+1}^{\infty}\frac{1}{ ( n+1 ) ^{p}} \Biggl\{ \Biggl[ \sum_{k=0}^{n}t^{ ( n-k ) q} \Biggr] ^{\frac{1}{q}} \Biggl[ \sum_{k=0}^{n} \vert x_{k} \vert ^{p} \Biggr] ^{\frac{1}{p}} \Biggr\} ^{p} \\ & \leq \Vert x \Vert _{p}^{p}\sum _{n=r+1}^{\infty}\frac{1}{ ( n+1 ) ^{p}} \Biggl[ \sum _{k=0}^{n}t^{ ( n-k ) q} \Biggr] ^{\frac{p}{q}} \\ & = \Vert x \Vert _{p}^{p}\sum _{n=r+1}^{\infty}\frac{1}{ ( n+1 ) ^{p}} \bigl[ 1+t^{q}+ \cdots+ \bigl( t^{q} \bigr) ^{n} \bigr] ^{\frac{p}{q}} \\ & = \Vert x \Vert _{p}^{p}\sum _{n=r+1}^{\infty}\frac{1}{ ( n+1 ) ^{p}} \biggl[ \frac{1- ( t^{q} ) ^{n+1}}{1-t^{q}} \biggr] ^{\frac{p}{q}}.\end{aligned} $$

Then we get

3.2 $$ \bigl\Vert A_{t}^{r}-A_{t} \bigr\Vert _{p}^{p}\leq \sum_{n=r+1}^{\infty} \frac{1}{ ( n+1 ) ^{p}} \biggl[ \frac{1- ( t^{q} ) ^{n+1}}{1-t^{q}} \biggr] ^{\frac{p}{q}}. $$

Hence, we obtain

$$ \frac{c_{n+1}}{c_{n}}=\frac{ ( n+1 ) ^{p}}{ ( n+2 ) ^{p}} \biggl[ \frac{1- ( t^{q} ) ^{n+2}}{1- ( t^{q} ) ^{n+1}} \biggr] ^{\frac{p}{q}}\rightarrow1, $$

where

$$ c_{n}=\frac{1}{ ( n+1 ) ^{p}} \biggl[ \frac{1- ( t^{q} ) ^{n+1}}{1-t^{q}} \biggr] ^{\frac{p}{q}}. $$

After that, we get

$$ \begin{gathered} n \biggl( \frac{c_{n+1}}{c_{n}}-1 \biggr)\\ \quad =n \biggl\{ \frac{ ( n+1 ) ^{p}}{ ( n+2 ) ^{p}} \biggl[ \frac{1- ( t^{q} ) ^{n+2}}{1- ( t^{q} ) ^{n+1}} \biggr] ^{\frac{p}{q}}-1 \biggr\} ,\quad t^{q}=:\beta \\ \quad =n \biggl\{ \biggl( 1-\frac{1}{n+2} \biggr) ^{p} \biggl[ 1- \frac{\beta ^{n+1}-\beta^{n+2}}{1-\beta^{n+1}} \biggr] ^{\frac{p}{q}}-1 \biggr\} \\ \quad =n \biggl\{ \biggl[ 1-\frac{p}{n+2}+o \biggl( \frac{1}{n+2} \biggr) \biggr] \biggl[ 1+\frac{p}{q}\frac{\beta^{n+1}-\beta^{n+2}}{1-\beta^{n+1}}+o \biggl( \frac{\beta^{n+1}-\beta^{n+2}}{1-\beta^{n+1}} \biggr) \biggr] -1 \biggr\} ,\end{gathered} $$

that is,

$$ n \biggl( \frac{c_{n+1}}{c_{n}}-1 \biggr) \rightarrow-p< -1. $$

Thus, from the Raabe test, $\sum_{n=0}^{\infty}c_{n}$ converges, and therefore $\sum_{k=n}^{\infty}c_{k}\rightarrow0$ (for $n\rightarrow\infty$). From (3.1), we have $\Vert A_{t}^{r}-A_{t} \Vert \rightarrow0$ (for $r\rightarrow\infty $). Thus, $A_{t}$ is the compact linear operator over $\ell_{p}$ ($1< p<\infty $) for $0< t<1$ from Theorem 5. □

## Spectrum of discrete generalized Cesàro operator

### Definition 2

Let $X\neq \{ 0 \} $ be a complex normed space and $T:D ( T ) \rightarrow X$ be a linear operator with domain $D ( T ) \subset X$. A number $\lambda\in \mathbb{C} $ that provides the following conditions is called the regular value of *T*, and the set of all regular values of *T* will be denoted by $\rho ( T ) $ and it is called the resolvent set of *T*:

1. $R_{\lambda} ( T ) :=T_{\lambda}^{-1}:= ( T-\lambda I ) ^{-1}$ resolvent operator exists,
2. $R_{\lambda} ( T ) $ is bounded, and
3. $R_{\lambda} ( T ) $ is defined on a set which is dense in X.

Moreover, $\sigma ( T ) =\mathbb{C} -\rho ( T ) $ is called the spectrum of *T*.

Furthermore, the spectrum $\sigma ( T ) $ naturally splits into three disjoint sets, some of which may be empty. The discrete splitting of the spectrum can be defined as the point spectrum, the continuous spectrum and the residual spectrum as follows.

### Definition 3

[4]

- The point spectrum or discrete spectrum $\sigma_{p} ( T ) $ is the set such that $R_{\lambda} ( T ) $ does not exist. A $\lambda \in\sigma_{p} ( T ) $ is called an eigenvalue of *T*.
- The continuous spectrum $\sigma_{c} ( T ) $ is the set such that $R_{\lambda} ( T ) $ exists and satisfies (R3) but not (R2), that is, $R_{\lambda} ( T ) $ is unbounded.
- The residual spectrum $\sigma_{r} ( T ) $ is the set such that $R_{\lambda} ( T ) $ exists (and may be bounded or not) but does not satisfy (R3), that is, the domain of $R_{\lambda} ( T ) $ is not dense in *X*.

Spectral theory is an important part of functional analysis. It plays a crucial role in many branches of mathematics such as function theory, complex analysis, differential and integral equations, control theory and also in numerous applications as they are intimately related to the stability of the underlying physical systems. For more information on spectrum, see [4].

The following theorem tells us that the point spectrum of a compact linear operator is not complicated. In fact, we also know that each spectral value $\lambda\neq0$ of a compact linear operator is an eigenvalue from the next theorem. The spectrum of a compact linear operator largely resembles the spectrum of an operator on a finite dimensional space.

### Theorem 7

[4]

*A compact linear operator* $T:X\rightarrow X$ *on a normed space* *X* *has the following properties*:

- *The set of the eigenvalues of T is countable* (*perhaps finite or even empty*).
- $\lambda=0$ *is the only possible point of accumulation of that set*.
- *Every spectral value* $\lambda\neq0$ *is an eigenvalue*.
- *If* *X* *is infinite dimensional*, *then* $0\in\sigma ( T ) $.

### Spectrum of discrete generalized Cesàro operator on $\ell _{p}$ ($1< p<\infty $)

Spectrum of compact Rhaly operator was specified in [5] and [6]. The spectrum of discrete generalized Cesàro operator $A_{t}$ on the Hilbert space $\ell_{2}$ was examined by Rhaly [1] in 1982. We determine the spectrum of $A_{t}$ on $\ell_{p}$ ($1< p<\infty $). Let $S:= \{ \frac{1}{n}:n=1,2,\ldots \} $.

In this section, we will compute the spectrum of the generalized discrete generalized Cesàro matrix, the compact linear operator $A_{t}$, where $0< t<1$.

#### Theorem 8

$\sigma_{p} ( A_{t},\ell _{p} ) =S$ *for* $0< t<1$, *where* $1< p<\infty$.

#### Proof

Let

$$ A_{t}x=\lambda x\quad\text{for }1< p< \infty, $$

where $x\neq\theta$. In this case, equations

4.1 $$ \begin{gathered} x_{0} =\lambda x_{0}, \\ \frac{1}{2} ( tx_{0}+x_{1} ) =\lambda x_{1}, \\ \frac{1}{3} \bigl( t^{2}x_{0}+tx_{1}+x_{2} \bigr) =\lambda x_{2}, \\ \frac{1}{4} \bigl( t^{3}x_{0}+t^{2}x_{1}+tx_{2}+x_{3} \bigr) =\lambda x_{3}, \\ \vdots \\ \frac{1}{n+1} \Biggl( \sum_{k=0}^{n}t^{n-k}x_{k} \Biggr) =\lambda x_{n}, \\ \vdots \end{gathered} $$

are provided.

(i) From equation $( 1-\lambda ) x_{0}=0$, if $x_{0}\neq0$ then $\lambda=1$. From (4.1), we have

$$ \begin{aligned} &\Rightarrow\quad \frac{1}{2} ( tx_{0}+x_{1} ) =x_{1} &{\Rightarrow}&\quad \frac{1}{2}tx_{0}=\frac{1}{2}x_{1} & \Rightarrow& \quad x_{1}=tx_{0} \\ &\Rightarrow \quad\frac{1}{3} ( t^{2}x_{0}+tx_{1}+x_{2} ) =x_{2} &{\Rightarrow}&\quad \frac{2}{3}t^{2}x_{0}=\frac{2}{3}x_{2} & \Rightarrow&\quad x_{2}=t^{2}x_{0} \\ &\Rightarrow & && \Rightarrow&\quad x_{n}=t^{n}x_{0},\quad x_{0}\neq0,0< t< 1 \\ & & &\vdots&& \end{aligned} $$

Since

$$ \biggl\vert \frac{x_{n+1}}{x_{n}} \biggr\vert ^{p}=t^{p} \rightarrow t^{p}< 1, $$

we get $\sum_{k} \vert x_{k} \vert ^{p}= \vert x_{0} \vert \sum_{k} \vert t^{p} \vert ^{k}<\infty$. Hence, we have $( x_{n} ) = ( t^{n}x_{0} ) \in\ell_{p}$. Therefore, the eigenvector corresponding to $\lambda=1$, $x= ( x_{0},tx_{0},t^{2}x_{0},t^{3}x_{0},\ldots ) \in\ell_{p}$, that is, we have $\lambda=1\in\sigma_{p} ( A_{t},\ell_{p} ) $.

(ii) Let $x_{0}=0$. Therefore, we obtain

$$ \frac{1}{2}x_{1}=\lambda x_{1}\quad\Rightarrow \quad\biggl( \lambda-\frac {1}{2} \biggr) x_{1}=0 $$

from the second equation in (4.1). If $x_{1}\neq0$, then $\lambda =\frac{1}{2}$. Hence, we obtain

$$ \begin{aligned} &\frac{1}{3} ( tx_{1}+x_{2} ) =\frac{1}{2}x_{2} & \Rightarrow&\quad \frac{1}{3}tx_{1}=\frac{1}{6}x_{2} & \Rightarrow\quad& x_{2}=2tx_{1}, \\ &\frac{1}{4} ( t^{2}x_{1}+tx_{2}+x_{3} ) =\frac{1}{2}x_{3} & \Rightarrow&\quad\frac{4}{3}t^{2}x_{1}=\frac{1}{4}x_{3} & \Rightarrow\quad& x_{3}=3t^{2}x_{1}, \\ &\frac{1}{5} ( t^{3}x_{1}+t^{2}x_{2}+tx_{3}+x_{4} ) =\frac{1}{2}x_{4} & \Rightarrow&\quad\frac{6}{5}t^{3}x_{1}=\frac{3}{10}x_{4} & \Rightarrow\quad& x_{4}=4t^{3}x_{1}, \\ & &&\vdots& \Rightarrow\quad &x_{n}=nt^{n-1}x_{1}\end{aligned} $$

from the other equations in (4.1). Then, since

$$ \biggl\vert \frac{x_{n+1}}{x_{n}} \biggr\vert ^{p}= \biggl( \frac {n+1}{n} \biggr) ^{p}t^{p}\rightarrow t^{p}< 1, $$

we have $\sum_{n} \vert x_{n} \vert ^{p}<\infty$, that is, $x= ( x_{n} ) \in\ell_{p}$. Thus, the eigenvector corresponding to $\lambda=\frac{1}{2}$ is $x= ( 0,x_{1},2tx_{1},3t^{2}x_{1},\ldots ) \in\ell_{p}$, *i.e*., $\lambda =1/2\in\sigma_{p} ( A_{t},\ell_{p} ) $.

(iii) If $x_{m}$ is the first nonzero component of the sequence $x= ( x_{n} ) $, then from *m*th equation in (4.1), *i.e*.,

$$ \frac{1}{m+1} \Biggl( \sum_{k=0}^{m}t^{m-k}x_{k} \Biggr) =\lambda x_{m}, $$

we get

$$ \frac{1}{m+1}x_{m}=\lambda x_{m}\quad\Rightarrow \quad\biggl( \lambda-\frac {1}{m+1} \biggr) x_{m}=0,\quad x_{m} \neq0\quad\Rightarrow\quad\lambda=\frac{1}{m+1}. $$

In this case, we have

$$ x_{m+n}=\frac{ ( m+1 ) ( m+2 ) \cdots ( m+n ) }{n!}t^{n}x_{m}\quad\text{for all }n\geq1 $$

from other equations in (4.1). Since $t\in ( 0,1 ) $,

$$ \biggl\vert \frac{x_{m+n+1}}{x_{m+n}} \biggr\vert ^{p}= \biggl( \frac {m+n+1}{n} \biggr) ^{p}t^{p}\rightarrow t^{p}< 1\quad (\text{by }n\rightarrow\infty),$$

the eigenvector corresponding to $\lambda=1/ ( m+1 ) $ is

$$ \begin{aligned} x ={}& \biggl( 0,0,\ldots,x_{m}, ( m+1 ) tx_{m},\frac{ ( m+1 ) ( m+2 ) }{2}t^{2}x_{m}, \\ & \ldots,\frac{ ( m+1 ) ( m+2 ) \cdots ( m+n ) }{n!}t^{n}x_{m},\ldots \biggr) \in \ell_{p},\end{aligned} $$

*i.e*., $\lambda=1/ ( m+1 ) \in\sigma_{p} ( A_{t},\ell _{p} ) $. Hence, $\sigma_{p} ( A_{t},\ell_{p} ) =S= \{ \frac{1}{m}:m=1,2,\ldots \} $. □

We will use the following lemma to find the adjoint on the $\ell_{p}$ ($1< p<\infty$) sequence space of a linear transform.

#### Lemma 1

[7], p. 215

*If* $A\in B ( \ell _{p} ) $ ($1< p<\infty$), *then* *A* *can be represented by an infinite matrix and* $A^{\ast}$, *which is an element of* $B ( \ell_{q} )$, *where* $\frac{1}{p}+\frac{1}{q}=1$, *can be represented by the transpose of* *A* *matrix*.

The adjoint matrix of $A_{t}$ on $\ell_{p}$ ($1< p<\infty$) is as follows:

#### Lemma 2

*The adjoint operator over* $\ell _{p} $ ($p>1 $) *of the matrix* $A_{t}$ *can be given as its transposition*. *That is*, *the matrix* $( A_{t} ) ^{\ast}= ( a_{nk}^{\ast} ) $ *is given by*

4.2 $$ a_{nk}^{\ast}= \left\{ \textstyle\begin{array}{l@{\quad}l} \frac{t^{k-n}}{k+1}, & 0\leq n\leq k, \\ 0, & n>k.\end{array}\displaystyle \right . $$

#### Theorem 9

$\sigma_{p} ( A_{t}^{\ast },\ell_{p}^{\ast}\cong\ell_{q} ) =S$ *for* $0< t<1$, *where* $1< p<\infty$.

#### Proof

Let $x\neq\theta$ and $A_{t}^{\ast}x=\lambda x$. Then, for all $n\geq1$, the equations

$$ \begin{gathered} x_{0}+\frac{t}{2}x_{1}+ \frac{t^{2}}{3}x_{2}+\frac{t^{3}}{4}x_{3}+\cdots = \lambda x_{0}, \\ \frac{1}{2}x_{1}+\frac{t}{3}x_{2}+ \frac{t^{2}}{4}x_{3}+\cdots =\lambda x_{1}, \\ \frac{1}{3}x_{2}+\frac{t}{4}x_{3}+\cdots = \lambda x_{2}, \\ \frac{1}{4}x_{3}+\cdots =\lambda x_{3}, \\ \vdots \end{gathered} $$

are realized from Lemma 2. Therefore $0\notin \sigma_{p} ( A_{t}^{\ast},\ell_{q} ) $ because if $\lambda=0$ then $x_{n}=0$ for all $n=0,1,2,\ldots$ . Hence, we get

$$ x_{n}=\frac{1}{t^{n}}\frac{ ( \lambda-\frac{1}{n} ) ( \lambda -\frac{1}{n-1} ) \cdots ( \lambda-1 ) }{\lambda^{n}}x_{0},\quad x_{0}\neq0 $$

because $x\neq\theta$. That is, we have

$$ x_{n}=\frac{1}{t^{n}}\prod_{k=1}^{n} \biggl( 1-\frac{1}{k\lambda} \biggr) x_{0}\quad\text{for all }n \geq1, $$

where $x_{0}\neq0$. If $\lambda=\frac{1}{m}$ for an integer *m*, then we have $\sum_{n} \vert x_{n} \vert ^{q}<\infty$ because $x_{n}=0$ for every $n\geq m$, so that, $x= ( x_{n} ) \in\ell_{q}$ is obtained. Hence, we get $\lambda=\frac{1}{m}\in\sigma_{p} ( A_{t}^{\ast},\ell_{p}^{\ast}\cong\ell_{q} ) $ for all integers *m*. Let $\lambda\neq\frac{1}{m}$ for all integers *m*. Since

$$ \biggl\vert \frac{x_{n+1}}{x_{n}} \biggr\vert ^{q}= \frac{1}{t^{q}} \biggl\vert 1-\frac{1}{\lambda ( n+1 ) } \biggr\vert ^{q}\rightarrow\frac {1}{t^{q}}>1\quad ( n\rightarrow\infty ) $$

$\sum_{n} \vert x_{n} \vert ^{q}$ series is divergent. So, there is no other eigenvalue, *i.e*., we have

$$ \sigma_{p} \bigl( A_{t}^{\ast},\ell_{q} \bigr) =S. $$

 □

#### Theorem 10

$\sigma ( A_{t},\ell _{p} ) =S\cup \{ 0 \} $ *for* $0< t<1$, *where* $1< p<\infty$.

#### Proof

Since $\dim\ell_{p}=\infty$, we have $0\in\sigma ( A_{t},\ell _{p} ) $ from Theorem 7. Also, since $A_{t}$ is a compact linear operator by Theorem 6, each nonzero spectral value of $A_{t}$ is an eigenvalue from Theorem 7. Therefore, $\sigma ( A_{t},\ell_{p} ) =S\cup \{ 0 \} $ is obtained from Theorem 8. □

### The fine spectrum of discrete generalized Cesàro operator on $\ell_{p}$ ($1< p<\infty $)

Let *X* be a Banach space, $B(X)$ denotes the collection of all bounded linear operators on *X* and $T\in B(X)$. Then there are three possibilities for $R ( T ) $, the range of *T*:

(I) $R ( T ) =X$,
(II) $\overline{R ( T ) }=X$, but $R ( T ) \neq X$,
(III) $\overline{R ( T ) }\neq X$,

and three possibilities for $T^{-1}$:

1. $T^{-1}$ exists and is continuous,
2. $T^{-1}$ exists but is discontinuous,
3. $T^{-1}$ does not exist.

If these possibilities are combined in all possible ways, nine different states are created. These are labeled by $\mathrm{I}_{1}$, $\mathrm{I}_{2}$, $\mathrm{I}_{3}$, $\mathrm{II}_{1} $, $\mathrm{II}_{2}$, $\mathrm{II}_{3}$, $\mathrm{III}_{1}$, $\mathrm{III}_{2}$, $\mathrm{III}_{3}$. For example, let an operator be in state $\mathrm{III}_{2}$. Then $\overline{R ( T ) }\neq X$ and $T^{-1}$ exist and $T^{-1}$ is unbounded. From the closed graph theorem, $I_{2}$ is empty (see [8]).

Applying the Goldberg classification to the operator $T_{\lambda }:=\lambda I-T $, we have

(I) $T_{\lambda}=\lambda I-T$ is surjective,
(II) $\overline{R(T_{\lambda})}=X$, but $R(T_{\lambda})\neq X$,
(III) $\overline{R(T_{\lambda})}\neq X$,

and three possibilities for $T_{\lambda}^{-1}$:

1. $T_{\lambda}=\lambda I-T$ is injective and $T_{\lambda }^{-1}$ is bounded,
2. $T_{\lambda}=\lambda I-T$ is injective and $T_{\lambda }^{-1}$ is unbounded, and
3. $T_{\lambda}=\lambda I-T$ is not injective.

If *λ* is a complex number such that $T_{\lambda}=\lambda I-T\in \mathrm{I}_{1}$ or $T_{\lambda}=\lambda I-T\in \mathrm{II}_{1}$, then $\lambda\in\rho (T,X)$. All scalar values of *λ* not in $\rho(T,X)$ comprise the spectrum of *T*. The further classification of $\sigma(T,X)$ gives rise to the fine spectrum of *T*. That is, $\sigma(T,X)$ can be divided into the subsets $\mathrm{I}_{2}\sigma(T,X)$, $\mathrm{I}_{3}\sigma(T,X)$, $\mathrm{II}_{2}\sigma(T,X)$, $\mathrm{II}_{3}\sigma (T,X)$, $\mathrm{III}_{1}\sigma(T,X)$, $\mathrm{III}_{2}\sigma(T,X)$, $\mathrm{III}_{3}\sigma(T,X)$. For example, if $T_{\lambda}=\lambda I-T$ is in a given state, $\mathrm{III}_{2}$ (say), then we write $\lambda\in \mathrm{III}_{2}\sigma(T,X)$.

We can summarize the above in Table 1.

**Table 1:** **Goldberg’s decomposition of the spectrum**

		**(1)**	**(2)**	**(3)**
		***R*** **(** ***λ*** **;** ***T*** **)** **exists and is bounded**	***R*** **(** ***λ*** **;** ***T*** **)** **exists and is unbounded**	***R*** **(** ***λ*** **;** ***T*** **)** **does not exists**
(I)	*R*(*λI* − *T*)=*X*	*λ*∈*ρ*(*T*)	-	$\lambda \in\sigma_{p} ( T ) $
(II)	$\overline{R(\lambda I-T)}=X$	*λ*∈*ρ*(*T*)	$\lambda\in\sigma_{c} ( T ) $	$\lambda\in\sigma_{p} ( T ) $
(III)	$\overline{R(\lambda I-T)}\neq X$	$\lambda\in\sigma _{r} ( T ) $	$\lambda\in\sigma_{r} ( T ) $	$\lambda\in \sigma _{p} ( T ) $

This classification of the spectrum is called the Goldberg classification. Let us give the theorems that will help the Goldberg classification.

#### Theorem 11

[8], p. 58

*If* $T^{\ast} $ *has a bounded inverse*, *then* $R ( T^{\ast} ) $ *is closed*.

#### Theorem 12

[8], p. 59

*T* *has a dense range if and only if* $T^{\ast}$ *is* 1-1.

#### Theorem 13

[8], p. 60

$R ( T^{\ast } ) =X^{\ast}$ *if and only if* *T* *has a bounded inverse*.

#### Theorem 14

[8], p. 60

$\overline{R ( T ) }=X$ *and* *T* *has a bounded inverse if and only if* $R ( T^{\ast } ) =X^{\ast}$ *and* $T^{\ast}$ *has a bounded inverse*.

The fine spectra of bounded linear operators defined by some particular limitation matrices over some sequence spaces were first discussed in [5, 9–11] and [12].

Then the spectra and fine spectra of some operators have been studied by various authors [13–22] and are still being studied.

We will examine the fine spectrum of a discrete generalized Cesàro operator on $\ell_{p}$ ($1< p<\infty $), which is compact in this section.

#### Theorem 15

$0\in \mathrm{II}_{2}\sigma ( A_{t},\ell_{p} ) $ *for* $0< t<1$, *where* $1< p<\infty$.

#### Proof

Since $\sigma_{p} ( A_{t},\ell_{p} ) =S$, we have $0\notin \sigma _{p} ( A_{t},\ell_{p} ) $. Thus, $( A_{t} ) ^{-1}$ exists. Hence $A_{t}\in ( 1 ) \cup ( 2 ) $. The operator $A_{t}^{\ast}$ is 1-1 because $0\notin\sigma_{p} ( A_{t}^{\ast },\ell_{q} ) $. Hence, we have $\overline{R(A_{t})}=\ell_{p}$ from Theorem 12. If $A_{t}x=y$, we obtain

$$ y_{n}=\frac{1}{n+1}\sum_{k=0}^{n}t^{n-k}x_{k}. $$

Therefore, we get

$$ x_{0}=y_{0}\quad \text{and}\quad x_{n}= ( n+1 ) y_{n}-tny_{n-1} $$

from

$$ \begin{gathered} ( n+1 ) y_{n} =t^{n}x_{0}+t^{n-1}x_{1}+ \cdots +tx_{n-1}+x_{n}, \\ tny_{n-1} =t \bigl( t^{n-1}x_{0}+t^{n-2}x_{1}+ \cdots+x_{n-1} \bigr) .\end{gathered} $$

Then we give the matrix $A_{t}^{-1}= ( c_{nk} ) $ with

$$ c_{nk}= \left\{ \textstyle\begin{array}{l@{\quad}l} n+1, & k=n, \\ -tn, & k=n-1, \\ 0, & \text{otherwise}.\end{array}\displaystyle \right . $$

If we take $y= ( y_{n} ) = ( \frac{ ( -1 ) ^{n}}{n+1} ) \in\ell_{p}$ ($1< p<\infty$), then we have

$$ ( x_{n} ) = \biggl( ( n+1 ) \frac{ ( -1 ) ^{n}}{n+1}- ( -1 ) ^{n-1} \frac{nt}{n} \biggr) = \bigl( ( -1 ) ^{n} ( 1+t ) \bigr) \notin \ell_{p}. $$

Hence $A_{t}$ is not onto, that is, $R(A_{t})\neq\ell_{p}$. Therefore, $A_{t}\in \mathrm{II}$. As a consequence, $A_{t}\in \mathrm{II}_{1}$ or $A_{t}\in \mathrm{II}_{2}$. We have $A_{t}\notin \mathrm{II}_{1}$ because $0\in\sigma ( A_{t},\ell _{p} ) $. Then we have $A_{t}\in \mathrm{II}_{2}$, *i.e*., $0\in \mathrm{II}_{2}\sigma ( A_{t},\ell_{p} ) $. □

#### Theorem 16

$\mathrm{III}_{3}\sigma ( A_{t},\ell_{p} ) =S$ *for* $0< t<1$, *where* $1< p<\infty$.

#### Proof

If $\lambda=\frac{1}{m}$, then $T_{\lambda}= ( \lambda I-A_{t} ) $ has no inverse because $\sigma_{p} ( A_{t},\ell_{p} ) =S= \{ \frac{1}{m}:m=1,2,\ldots \} $, that is, we have $T_{\lambda}\in ( 3 ) $. Since $\lambda=\frac{1}{m}\in\sigma_{p} ( A_{t}^{\ast},\ell_{p} ) $, operator $T_{\lambda}^{\ast}=\lambda I-A_{t}^{\ast}$ is not 1-1 for $\lambda=\frac{1}{m}$. $T_{\lambda }=\lambda I-A_{t}$ does not have a dense image by Theorem 12. Hence, $\overline{R ( T_{\lambda} ) }\neq\ell_{p}$, *i.e*., $T_{\lambda}\in \mathrm{III}$. Accordingly, $T_{\frac{1}{m}}=\frac{1}{m}I-A_{t}\in \mathrm{III}_{3}$, and hence, we have $\lambda=\frac{1}{m}\in \mathrm{III}_{3}\sigma ( A_{t},\ell_{p} ) $. □

## Subdivision of the spectrum of discrete generalized Cesàro operator on $\ell_{p}$ ($1< p<\infty $)

Given a bounded linear operator *T* in a Banach space *X*, we call a sequence $(x_{k})$ in *X* a Weyl sequence for *T* if $\Vert x_{k} \Vert =1$ and $\Vert Tx_{k} \Vert \rightarrow0$ as $k\rightarrow\infty$.

In what follows, we call the set

5.1 $$ \sigma_{ap} ( T ) :=\{\lambda\in\mathbb{K}:\text{there exists a Weyl sequence for }\lambda I-T\} $$

the approximate point spectrum of *T*. Moreover, the subspectrum

5.2 $$ \sigma_{\delta} ( T ) :=\{\lambda\in\mathbb{K}:\lambda I-T\text{ is not surjective}\} $$

is called the defect spectrum of *T*.

The two subspectra (5.1) and (5.2) form a (not necessarily disjoint) subdivision

5.3 $$ \sigma ( T ) =\sigma_{ap} ( T ) \cup\sigma_{\delta } ( T ) $$

of the spectrum. There is another subspectrum

5.4 $$ \sigma_{co} ( T ) = \bigl\{ \lambda\in\mathbb{K}:\overline {R(\lambda I-T)}\neq X \bigr\} $$

which is often called compression spectrum in the literature and which gives rise to another (not necessarily disjoint) decomposition

5.5 $$ \sigma ( T ) =\sigma_{ap} ( T ) \cup\sigma _{co} ( T ) $$

of the spectrum. Clearly, $\sigma_{p} ( T ) \subseteq\sigma _{ap} ( T ) $ and $\sigma_{co} ( T ) \subseteq\sigma _{\delta} ( T ) $. Moreover, comparing these subspectra, we note that

5.6 $$ \sigma_{r} ( T ) =\sigma_{co} ( T ) \setminus \sigma _{p} ( T ) $$

and

5.7 $$ \sigma_{c} ( T ) =\sigma ( T ) \setminus \bigl[ \sigma _{p} ( T ) \cup\sigma_{co} ( T ) \bigr]. $$

It can sometimes be useful to establish a relationship between the spectra of a bounded linear operator and its adjoint.

### Proposition 1

[23], Proposition 1.3

*The spectra and subspectra of an operator* $T\in B(X)$ *and its adjoint* $T^{\ast}\in B(X^{\ast})$ *are related by the following relations*:

- $\sigma_{ap} ( T^{\ast} ) =\sigma_{\delta} ( T ) $;
- $\sigma_{\delta} ( T^{\ast} ) =\sigma_{ap} ( T ) $;
- $\sigma_{p} ( T^{\ast} ) =\sigma_{co} ( T ) $;
- $\sigma ( T ) =\sigma_{ap} ( T ) \cup \sigma _{p} ( T^{\ast} ) =\sigma_{p} ( T ) \cup\sigma _{ap} ( T^{\ast} ) $.

By the definitions given above, we can write Table 2.

**Table 2:** **Separations of the spectrum [** 24 **]**

		**(1)**	**(2)**	**(3)**
		***R*** **(** ***λ*** **;** ***T*** **)** **exists and is bounded**	***R*** **(** ***λ*** **;** ***T*** **)** **exists and is unbounded**	***R*** **(** ***λ*** **;** ***T*** **)** **does not exists**
(I)	*R*(*λI* − *T*)=*X*	*λ*∈*ρ*(*T*)	-	$\lambda\in\sigma_{p} ( T ) $ $\lambda\in\sigma_{ap} ( T ) $
(II)	*R*(*λI* − *T*)≠*X* $\overline {R ( \lambda I-T ) }=X$	*λ*∈*ρ*(*T*)	$\lambda\in\sigma_{c} (T ) $ $\lambda\in\sigma _{ap} ( T ) $ $\lambda\in\sigma_{\delta} ( T ) $	$\lambda\in\sigma_{p} ( T ) $ $\lambda \in\sigma_{ap} ( T ) $ $\lambda\in\sigma_{\delta } ( T ) $
(III)	$\overline{R ( \lambda I-T ) }\neq X$	$\lambda\in\sigma_{r} ( T ) $ $\lambda\in\sigma _{\delta} ( T ) $ $\lambda\in\sigma_{co} ( T ) $	$\lambda\in\sigma_{r} ( T ) $ $\lambda \in\sigma_{ap} ( T ) $ $\lambda\in\sigma_{\delta } ( T ) $ $\lambda\in\sigma_{co} ( T ) $	$\lambda\in\sigma_{p} ( T ) $ $\lambda\in\sigma _{ap} ( T ) $ $\lambda\in\sigma_{\delta} ( T ) $ $\lambda\in\sigma_{co} ( T ) $

This separation of the spectrum of some operator has been studied by various authors in [18, 24–26, 28] and is still being studied.

### Theorem 17

*For* $0< t<1$ *and* $1< p<\infty$, *we have*

- $\sigma_{ap} ( A_{t},\ell_{p} ) =S\cup \{ 0 \}$;
- $\sigma_{\delta} ( A_{t},\ell_{p} ) =S\cup \{ 0 \}$;
- $\sigma_{co} ( A_{t},\ell_{p} ) =S$.

### Proof

(a) We have $\mathrm{III}_{1}\sigma ( A_{t},\ell_{p} ) =\emptyset$ from Table 2 because $\sigma ( A_{t},\ell_{p} ) =S\cup \{ 0 \} $ by Theorem 10, $\mathrm{III}_{3}\sigma ( A_{t},\ell_{p} ) =S$ by Theorem 16 and $\mathrm{II}_{2}\sigma ( A_{t},\ell_{p} ) = \{ 0 \} $ by Theorem 15. Hence, we get

$$ \sigma_{ap} ( A_{t},\ell_{p} ) =\sigma ( A_{t},\ell _{p} ) \setminus \mathrm{III}_{1}\sigma ( A_{t},\ell_{p} ) =S\cup \{ 0 \} $$

by Table 2.

(b) Since $\sigma ( A_{t},\ell_{p} ) =S\cup \{ 0 \} $, $\mathrm{III}_{3}\sigma ( A_{t},\ell_{p} ) =S$ and $\mathrm{II}_{2}\sigma ( A_{t},\ell_{p} ) = \{ 0 \} $ from respectively Theorems 10, 16 and 15, we have $\mathrm{I}_{3}\sigma ( A_{t},\ell _{p} ) =\emptyset$ by Table 2. Therefore, we obtain

$$ \sigma_{\delta} ( A_{t},\ell_{p} ) =\sigma ( A_{t},\ell _{p} ) \setminus \mathrm{I}_{3}\sigma ( A_{t},\ell_{p} ) =S\cup \{ 0 \} $$

by Table 2.

(c) Since $\sigma ( A_{t},\ell_{p} ) =S\cup \{ 0 \} $, $\mathrm{III}_{3}\sigma ( A_{t},\ell_{p} ) =S$ and $\mathrm{II}_{2}\sigma ( A_{t},\ell_{p} ) = \{ 0 \} $ from Theorems 10, 16 and 15 respectively, we obtain $\mathrm{III}_{1}\sigma ( A_{t},\ell _{p} ) =\emptyset$ from Table 2. As a result,

$$ \sigma_{co} ( A_{t},\ell_{p} ) =\mathrm{III}_{1}\sigma ( A_{t},\ell _{p} ) \cup \mathrm{III}_{2}\sigma ( A_{t},\ell_{p} ) \cup \mathrm{III}_{3}\sigma ( A_{t},\ell_{p} ) =S $$

by Table 2. □

### Lemma 3

*For* $0< t<1$ *and* $1< p<\infty$, *we have*

- $\sigma_{ap} ( A_{t}^{\ast},\ell_{q} ) =S\cup \{ 0 \}$;
- $\sigma_{\delta} ( A_{t}^{\ast},\ell_{q} ) =S\cup \{ 0 \} $.

### Proof

Since $\sigma_{ap} ( A_{t}^{\ast},\ell_{q} ) =\sigma _{\delta } ( A_{t},\ell_{p} ) $ and $\sigma_{\delta} ( A_{t}^{\ast },\ell_{q} ) =\sigma_{ap} ( A_{t},\ell_{p} ) $ from Proposition 1, the proof is clear. □

## Conclusions

The spectra of summability methods, the Goldberg classification of the spectrum and the non-discrete spectral separation of this summability methods were discussed by various authors earlier. Still, a lot of mathematicians work on this subject. The spectrum of the discrete generalized Cesàro operator on a Hilbert space $\ell_{2}$ was calculated by Rhaly [1] in 1982. In this article, we have obtained the spectra and various spectral separations of this operator over $\ell_{p}$ ($1< p<\infty$) sequence spaces. In [27], Yildirim *et al*. gave the spectra and spectral division of this operator over the $c_{0}$ and *c* sequence spaces. Also, a Mercerian theorem was given in [27]. The spectra and spectral separation of this operator over the other sequence spaces are left as clear problems.
